# The role of partners’ educational attainment in the association between HIV and education amongst women in seven sub-Saharan African countries

**DOI:** 10.7448/IAS.19.1.20038

**Published:** 2016-02-19

**Authors:** Guy Harling, Till Bärnighausen

**Affiliations:** 1 Department of Global Health and Population, Harvard T.H. Chan School of Public Health Boston, MA, USA; 2 Africa Centre for Population Health, Mtubatuba, KwaZulu-Natal, South Africa

**Keywords:** sub-Saharan Africa, education, sexual partnerships, homophily, HIV, partner characteristics

## Abstract

**Introduction:**

Individuals’ educational attainment has long been considered as a risk factor for HIV. However, little attention has been paid to the association between partner educational attainment and HIV infection.

**Methods:**

We conducted cross-sectional analysis of young women (aged 15–34) in 14 Demographic and Health Surveys from seven sub-Saharan Africa (SSA) countries with generalized HIV epidemics. We measured the degree of similarity in educational attainment (partner homophily) in 75,373 partnerships and evaluated the correlation between homophily and female HIV prevalence at the survey cluster level. We then used logistic regression to assess whether own and partner educational attainment was associated with HIV serostatus amongst 38,791 women.

**Results:**

Educational attainment was positively correlated within partnerships in both urban and rural areas of every survey (Newman assortativity coefficients between 0.09 and 0.44), but this correlation was not ecologically associated with HIV prevalence. At the individual level, larger absolute differences between own and partner educational attainment were associated with significantly higher HIV prevalence amongst women. This association was heterogeneous across countries, but not between survey waves. In contrast to other women, for those aged 25–34 who had secondary or higher education, a more-educated partner was associated with lower HIV prevalence.

**Conclusions:**

HIV prevalence amongst women in SSA is associated not only with one's own education but also with that of one's partner. These findings highlight the importance of understanding how partners place individuals at risk of infection and suggest that HIV prevention efforts may benefit from considering partner characteristics.

## Introduction

Even in the context of a generalized HIV epidemic in sub-Saharan Africa (SSA), the risk of infection varies greatly within small geographic or social groupings [1–3]. As a result, there is considerable interest in identifying those at increased risk of infection, who can then targeted for interventions [4,5]. One characteristic often thought to predict the risk of HIV infection is educational attainment. The absence of a higher risk of HIV infection for individuals with low socioeconomic status was one of the more surprising findings of early research into predictors of HIV risk in SSA. This null, or even reverse, association was seen for both income and wealth [6,7] and education [8–11].

Several conceptual mechanisms might be expected to lead to the more-educated having *lower* risk of HIV infection. More-educated people typically have stronger sociocognitive abilities, leading to better ability to assimilate risk information (which they are more likely to learn at school) and higher self-efficacy to act on such knowledge. More-educated individuals also tend to have more income and thus more control over their lives and ability to act on knowledge; they also tend to place higher value on the future and thus be more motivated to take preventative measures [12,13]. Nevertheless, these advantages may be offset by factors driven by the greater wealth and mobility that education can bring, notably a greater ability to attract and maintain multiple partners, and greater access to risky sexual networks, including sex workers and other mobile individuals. Such behaviours are particularly risky early in a new epidemic, when preventative knowledge is in short supply [14].

There have been at least two approaches to understanding the empirical HIV–education association. One strand has focused on place, finding own education to be less harmful in urban compared with rural settings [15–18], and finding increased community-level education to be generally protective against HIV [15,19]. The other strand has focused on changes in HIV knowledge and behavioural adaptations to the HIV epidemic, and how the HIV–education relationship has changed dynamically over time. This latter approach has often been guided by the theory that an inversion of the education–HIV gradient may reflect more-educated individuals being able to more-rapidly learn prevention strategies and more-easily implement them [20]. These theories are supported by evidence that education has become less of an HIV risk factor, or even protective against HIV, over the past 20 years [14,20–24], due to more rapid improvements in preventative behaviours (such as partner numbers, condom use and age at marriage) by more-educated individuals [23,25,26]. While both these analytic approaches are important, neither deals directly with the issue of with whom one partners: since HIV infection in Africa is passed primarily through a sexual network, the riskiness of one's partner is likely to be central to one's own risk of infection.

One step towards understanding the socioeconomic structure of sexual networks is to consider the education level of one's partner. We might expect partners’ educational attainment to affect HIV risk through at least two channels. First, given an existing pattern of HIV prevalence by education level, one's partner's education is a predictor of the potential for infection within a relationship. Increased risk may arise because a partner is more likely to be infected at relationship initiation, or because a partner is more likely to become infected whilst the relationship is ongoing. For example, more-educated men are often thought to be more central within sexual networks because they have the resources to attract many women [27]. If these men then partner with less-educated women – who are those most in need of such resource-transferring relationships [28,29] – then such relationships would see more prevalent HIV than other relationships involving low-education women.

Second, given the above-outlined sociocognitive factors associated with increased education, one's partner's education is likely to affect sexual behaviour within the relationship, even after allowing for own education. Such an effect arises from the observation that sexual behaviour within a couple is determined through negotiation, and is thus a product of each partner's preferences (e.g. condom non-use, sexual activities with higher risk of tears and abrasions) and ability to adhere to these preferences within the relationship. The sexual behaviours of the same individual across multiple relationships may therefore vary depending on the preferences and relationship power of their partners. If more education is associated with less-risky behaviours, then we would expect a more-educated partner to be associated with lower HIV infection risk, particularly if the partner has more power within the relationship. As an additional benefit, more-knowledgeable partners may affect your subsequent behaviour in other relationships, if they pass on their knowledge either through discussion or example.

A body of research points to a worldwide tendency to partner with people with similar educational attainment (i.e., educational homophily) worldwide [30–33]. Evidence from SSA is more limited but still suggests assortative partnering [30,34–36]. In high-income settings, an association has previously been seen between spousal education and both health behaviours [37] and all-cause, self-reported and non-communicable disease mortality [38–42]. The effect of husband's education on mortality is often weaker – although often still significant – than that of wives; an additional association between husband's occupation and mortality in women has also been observed [43].

Evidence regarding partner education and HIV, however, is very limited. Elevated rates of partner mixing between high- and low-risk individuals, in combination with strong racial homophily, has previously been hypothesized to drive the much higher rates of HIV infection in African Americans, compared with compatriots in other racial groups [44,45]. A recent review of the association between partner characteristics and sexually transmitted infections (STI) globally [46] found that of the only three analyses of partner education and STI risk, two focused on bacterial STIs in the United States [47,48]. The one cross-sectional study of HIV in Africa found that amongst 15- to 26-year-old South African women, those with partners who had graduated from secondary school had almost double the adjusted odds of HIV infection [49].

Given this limited research into partner education as a risk factor for HIV, we investigated whether partner's education attainment is associated with HIV serostatus in SSA. We focused on young SSA women since they face the highest force of HIV infection in the world [50,51]. We hypothesized (i) that areas with greater educational homophily would have lower HIV prevalence due to more limited mixing between high- and low-prevalence subgroups, (ii) that women with more-educated partners would be at higher risk of being HIV positive but (iii) that this second effect would weaken with calendar time.

## Methods

For this analysis, we used data from 14 nationally representative Demographic and Health Surveys (DHS) conducted in seven countries: Cameroon in 2004 and 2011, Ethiopia in 2005 and 2011, Kenya in 2003 and 2008–2009, Lesotho in 2004 and 2009, Malawi in 2004 and 2010, Rwanda in 2005 and 2010 and Zimbabwe in 2005–2006 and 2010–2011. These seven countries represented all sub-Saharan nations in which (i) two DHS surveys had been conducted with linked HIV testing, (ii) HIV prevalence was over 5% in the sample and (iii) data were available before the end of 2014. Detailed sampling plans are available from survey final reports available at www.dhsprogram.com/publications. DHS employs a multistage stratified design: every survey is stratified by urban status and by country-specific geographic or administrative regions; within strata each household has an equal probability of selection for interviews. Women aged 15–49 are interviewed in each selected household. In a proportion of selected households, anonymous HIV testing is also conducted (proportions vary by survey, see Supplementary Table 1). We further restricted our analyses to women aged 15–34, in order to both focus on higher incidence age groups, and since the risk of reverse causation – HIV status determining partner education level – rises with age.

Our outcome was HIV seroprevalence measured using dried blood spots that were laboratory tested using two enzyme-linked immunosorbent assays (ELISA) and confirmatory Western Blots as needed [52]. The primary exposure measures were self-reported educational attainment and report of partner's educational attainment. (Partner reports closely matched men's report of their own education level: for the 22,536 women whose partners were also interviewed, the two reports were identical for 70.8% and correlated at ρ>0.92). Our sample was limited to women who were, or had been, in a marriage-like relationship, since the DHS only requests partner characteristics from such women. Although years of education have different meanings in different countries, since our primary analyses were stratified by survey location, we were able to make country-specific comparisons. We therefore used education as a continuous measure of attainment, since this allows for a finer-grained analysis than grouping by level of educational attainment.

We conducted two sets of analyses, both accounting for the complex DHS survey design by allowing for clustering at the level of the primary sampling unit (typically a village or census area). Our first analysis included all women in the 14 surveys who provided information on both their own and their partner's education level. For this sample, we calculated how assortative educational mixing was within each survey's region/urban strata, using Newman's assortativity coefficient, a variant of the Pearson correlation coefficient [53,54]. Newman assortativity coefficient values can range from –1 to 1, with a significant positive (negative) association indicating more matching of couples with similar (dissimilar) levels of education than would be expected by chance. We considered how assortativity varied with time, by geography and by stratum-specific measures of HIV prevalence and female educational attainment. For this analysis, we used the sample weights for the main survey provided by DHS.

For our second analysis, we removed from our first sample women without an HIV test result and used the HIV-specific sample weights provided by DHS. In the resulting dataset, we ran three logistic regression models for prevalent HIV infection, including country, urbanicity, woman's age (15–19; 20–24; 25–29; 30–35) and survey round as covariates. In model 1, we included only each woman's *educational attainment* in years. In model 2, we added the difference in years between each woman's husband's educational attainment and their own (*educational difference*), testing whether the model fit was improved using Wald tests. We further considered at each step whether any results from the pooled analysis varied across our sample. To do this, we added interaction terms for country and year with women's education (model 1) and educational difference (model 2). In model 3, we added the interaction of the educational difference and each woman's educational attainment level (none, primary, secondary or above) to determine whether any effect in the second model differed by how educated women were.

Finally, we considered whether any of our results differed when we stratified our sample into those aged under and over 25 years, under the hypotheses that a stronger positive association between partner education and HIV in the younger age range would relate to infection risk, whilst a stronger association in the older group would relate to longevity. In all cases, we included linear and quadratic terms for educational attainment and educational difference, to allow for non-linear associations with HIV prevalence.

A relevant national ethics review board reviews each DHS survey, and data collection procedures are approved by the Macro International institutional review board. Informed consent was gained for the surveys and for HIV testing. This study was exempted from additional ethical review by the Harvard T.H. Chan School of Public Health Institutional Review Board because of its use of anonymized secondary data.

## Results

Our sample for calculating partner assortativity comprised the 75,373 female respondents aged between 15 and 34 in the 14 surveys who provided both information on their own and their partner's education level. Our study sample for the HIV analysis was the 38,791 women from the above sample who in addition had a valid HIV test result (HIV test response rates ranged from 70 to 99%, see Supplementary Table 1). Descriptive statistics are provided in Table 1 (and stratified by country in Supplementary Table 2 for the ‘assortativity sample’ and Supplementary Table 3 for the ‘HIV sample’).

**Table 1:** Descriptive statistics for pooled data on women from 14 Demographic and Health Surveys

	Assortativity sample	HIV sample	
	
		*N*	% seropositive
All observations (*n*)	75,373	38,791	10.9
Urbanicity			
Non-urban	74.7	74.5	9.1
Urban	25.3	25.5	16.3
Own age			
15–19	10.5	10.6	4.8
20–24	29.8	29.3	8.4
25–29	33.2	33.5	12.0
30–34	26.4	26.6	14.8
Own education (years)^a^	6 [1–8]	6 [0–8]	
Male partner education (years)^a^	7 [2–10]	7 [2–10]	
Male–female educational difference (years)^a^	0 [0–3]	0 [0–3]	

The 14 Demographic and Health Surveys (DHS) included were: Cameroon: 2004, 2011; Ethiopia: 2005, 2011; Kenya: 2003, 2008–2009; Lesotho: 2004, 2009; Malawi: 2004, 2010; Rwanda: 2005, 2010; and Zimbabwe: 2005–2006, 2010–2011. Figures are proportions unless otherwise noted;

a Denotes medians and interquartile ranges. Proportions and percentiles are survey weighted using the DHS sample weights: for the Assortativity sample using female sample weights; for the HIV sample using HIV sample weights. Descriptive statistics for each survey provided in Supplementary Table 2 (assortativity sample) and Supplementary Table 3 (HIV sample).

Educational attainment and HIV prevalence varied widely across survey countries, with both being higher in urban than rural settings for all 14 surveys. Female education ranged from a low of 77.7% with no education and 1.7% with any secondary education in rural Ethiopia in 2005, to a high of 0.2% with none and 88.2% with some secondary education in urban Zimbabwe in the same year. Male partners’ education was highly correlated with female education level ρ>0.9 for the HIV dataset), and slightly higher than that of their female partners, except in Lesotho. HIV prevalence amongst women ranged from 1.8% in Ethiopia in 2005 to 32.5% in Lesotho in 2004.

**Figure 1:**
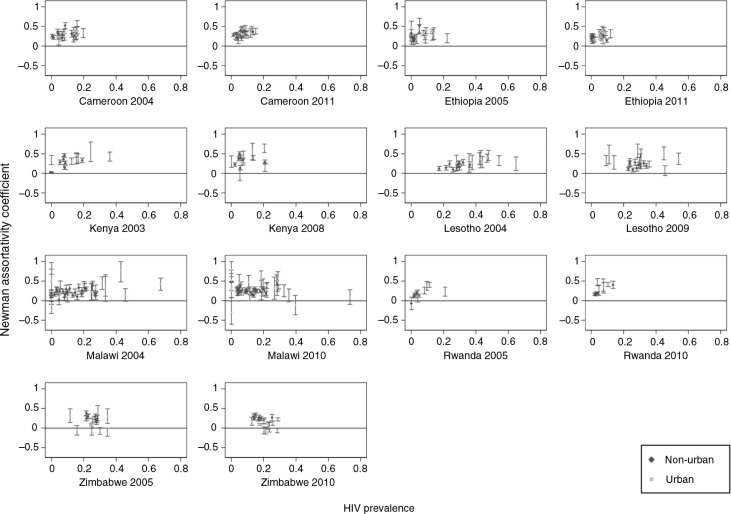
Newman assortativity coefficient for educational attainment within relationships and HIV prevalence. Partner-level educational assortativity was not correlated with female HIV prevalence (regionally, *n=*308: ρ=0.03, *p=*0.65; nationally, *n=*14: ρ=–0.16, *p=*0.41) using Pearson correlation coefficients. Sample size for education measures was 75,373; sample size for HIV prevalence was 38,791, weighted for the HIV sample.

Partner mixing was homophilous by education in all countries, settings and time periods. Country-level Newman coefficient's ranged from 0.09 (95% confidence interval: 0.07–0.11) in urban Zimbabwe to 0.44 (95% confidence interval: 0.42–0.46) in non-urban Cameroon (Table 2). Educational assortativity was not associated with female educational attainment (Supplementary Figure 1), and changed only slightly and non-systematically between survey rounds. The level of assortativity was not significantly associated with HIV prevalence across primary sampling units (ρ=0.03, *p*=0.65, *n=*308; Figure 1).

**Table 2:** Newman assortativity coefficients (and 95% confidence intervals) for educational mixing by attainment level

		First survey	Second survey
Cameroon	Non-urban	0.45 (0.43–0.47)	0.43 (0.41–0.45)
	Urban	0.40 (0.38–0.42)	0.40 (0.38–0.42)
Ethiopia	Non-urban	0.22 (0.20–0.23)	0.28 (0.26–0.29)
	Urban	0.35 (0.32–0.37)	0.31 (0.28–0.33)
Kenya	Non-urban	0.39 (0.37–0.41)	0.40 (0.38–0.42)
	Urban	0.40 (0.36–0.43)	0.33 (0.30–0.37)
Lesotho	Non-urban	0.20 (0.18–0.22)	0.23 (0.21–0.25)
	Urban	0.36 (0.31–0.41)	0.27 (0.22–0.31)
Malawi	Non-urban	0.22 (0.21–0.23)	0.27 (0.26–0.28)
	Urban	0.37 (0.33–0.41)	0.35 (0.32–0.38)
Rwanda	Non-urban	0.14 (0.12–0.16)	0.19 (0.17–0.20)
	Urban	0.35 (0.31–0.39)	0.44 (0.39–0.48)
Zimbabwe	Non-urban	0.30 (0.28–0.33)	0.27 (0.25–0.29)
	Urban	0.13 (0.11–0.15)	0.09 (0.08–0.11)

The 14 Demographic and Health Surveys (DHS) included were: Cameroon: 2004, 2011; Ethiopia: 2005, 2011; Kenya: 2003, 2008–2009; Lesotho: 2004, 2009; Malawi: 2004, 2010; Rwanda: 2005, 2010; and Zimbabwe: 2005–2006, 2010–2011. Total sample size was 75,373; sample sizes for each survey are provided in Supplementary Table 2.

**Figure 2:**
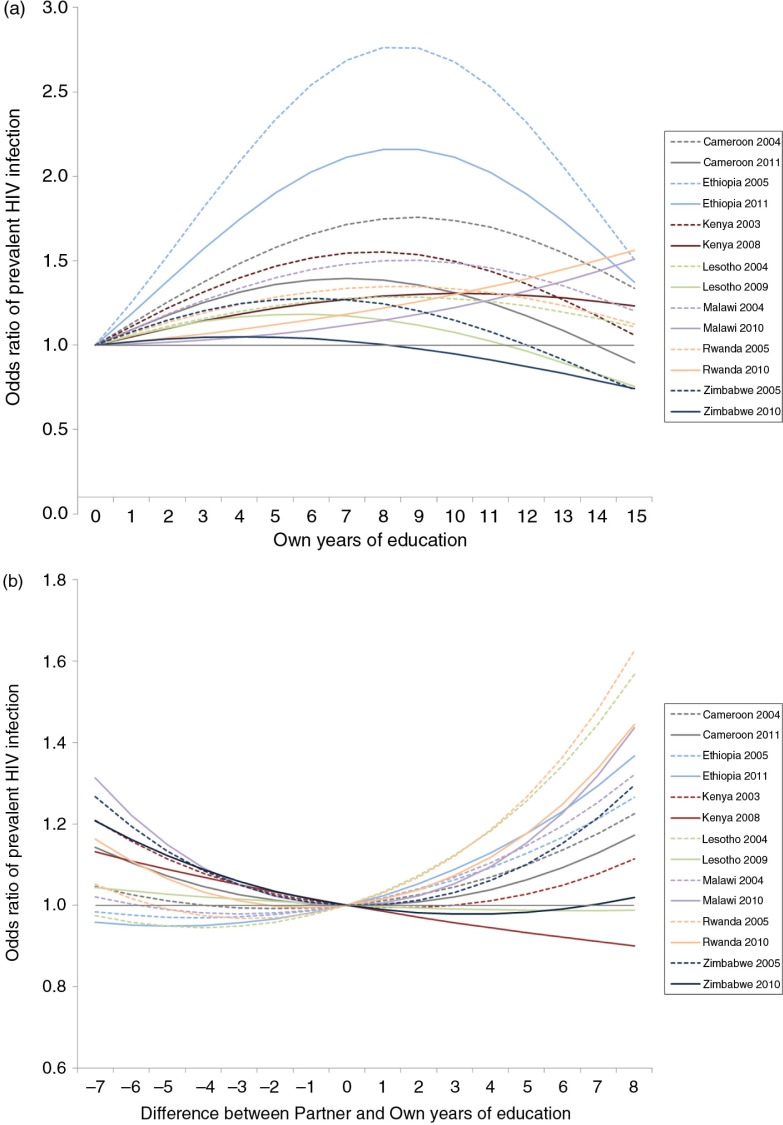
Association of own education and relationship educational difference on woman's risk of prevalent HIV infection, stratified by DHS survey. Odds ratios are based on regression coefficients from a single model containing interactions of: (i) women's education and DHS study and (ii) difference between partner and woman's education and DHS study; model also contains variables for urbanicity and woman's age. Odds ratios calculated by combining linear and quadratic variable coefficients for each variable shown, and thus are relative to a woman in the same survey with no education (panel a) or the same education as their partner (panel b).

In model 1, own education was, on average, associated with increased risk of HIV infection (Table 3). In an interaction model containing both country and survey round interaction, this effect was heterogeneous across countries (*F*_(12, 6424)_=6.06, *p*<0.001), often with risk rising with primary education, but then peaking and falling for higher levels of attainment (Figure 2a). In almost all countries, the relative odds of HIV infection fell for more-educated individuals between surveys, and overall this decline was significant (*F*_(2, 6434)_=5.85, *p=*0.003).

**Table 3:** Logistic regression models of HIV status on own and partner education in 14 DHS surveys

	Model 1		Model 2		Model 3		Model 3, <25 years		Model 3, >25 years	
Sample size		38,791		38,791		38,791		15,476		23,303
Cameroon	1.00		1.00		1.00		1.00		1.00	
Ethiopia	0.48	[0.37–0.63]	0.50	[0.39–0.66]	0.50	[0.38–0.65]	0.35	[0.22–0.57]	0.58	[0.42–0.78]
Kenya	1.47	[1.20–1.79]	1.46	[1.19–1.78]	1.38	[1.13–1.68]	1.78	[1.29–2.46]	1.25	[0.99–1.59]
Lesotho	6.18	[5.24–7.29]	6.27	[5.28–7.45]	6.07	[5.10–7.23]	5.60	[4.15–7.54]	6.32	[5.14–7.76]
Malawi	2.40	[2.03–2.84]	2.38	[2.02–2.82]	2.29	[1.93–2.71]	1.93	[1.43–2.59]	2.48	[2.04–3.02]
Rwanda	0.58	[0.48–0.71]	0.61	[0.50–0.74]	0.60	[0.49–0.73]	0.73	[0.48–1.10]	0.60	[0.48–0.75]
Zimbabwe	3.45	[2.97–4.02]	3.49	[3.01–4.05]	3.37	[2.91–3.92]	2.59	[2.01–3.33]	3.81	[3.19–4.56]
Urban vs. Rural	1.80	[1.62–2.01]	1.73	[1.55–1.93]	1.74	[1.56–1.94]	1.71	[1.43–2.04]	1.75	[1.54–1.99]
Second survey round	0.79	[0.72–0.86]	0.77	[0.71–0.85]	0.77	[0.70–0.85]	0.62	[0.53–0.73]	0.85	[0.76–0.94]
Own Age										
15–19	1.00		1.00		1.00		1.00			
20–24	1.81	[1.50–2.19]	1.82	[1.50–2.20]	1.82	[1.51–2.20]	1.83	[1.50–2.22]		
25–29	3.17	[2.64–3.81]	3.15	[2.62–3.79]	3.16	[2.62–3.80]			1.00	
30–35	4.00	[3.32–4.82]	3.97	[3.30–4.79]	3.99	[3.31–4.82]			1.28	[1.16–1.40]
Own education										
Years	1.20	[1.15–1.25]	1.24	[1.19–1.29]	1.27	[1.21–1.34]	1.27	[1.15–1.40]	1.28	[1.21–1.36]
Years squared	0.99	[0.98–0.99]	0.99	[0.98–0.99]	0.99	[0.98–0.99]	0.98	[0.98–0.99]	0.99	[0.98–0.99]
Relationship educational difference*										
All women										
Years			1.01	[1.00–1.03]						
Years squared			1.01	[1.01–1.01]						
Women with no education										
Years					1.05	[0.96–1.15]	1.00	[0.82–1.23]	1.06	[0.96–1.17]
Years squared					1.01	[1.00–1.01]	1.01	[0.99–1.03]	1.01	[1.00–1.01]
Women with primary education										
Years					1.03	[1.01–1.05]	1.02	[0.99–1.05]	1.03	[1.01–1.06]
Years squared					1.01	[1.00–1.01]	1.01	[1.00–1.01]	1.01	[1.00–1.01]
Women with secondary education and above										
Years					0.97	[0.95–1.00]	1.02	[0.97–1.06]	0.96	[0.93–0.99]
Years squared					1.00	[1.00–1.01]	1.01	[1.00–1.01]	1.00	[1.00–1.00]

All models take account of the clustered, non-self-weighting design of Demographic and Health Surveys. In the age-stratified models, 12 women were dropped to allow model convergence.

* Relationship educational difference measured as years of education of male partner minus years of education of respondent.

In model 2, greater educational difference was associated with increased risk of HIV infection; this effect was present whether the male partner was more educated or less educated. Again, this effect was slightly heterogeneous across countries (*F*_(12, 6424)_=1.86, *p=*0.03), but in this model, there was no clear change over time (Figure 2b; *F*_(2, 6434)_=1.31, *p=*0.27). The magnitude of association seen for educational difference was smaller than that seen for own education.

**Figure 3:**
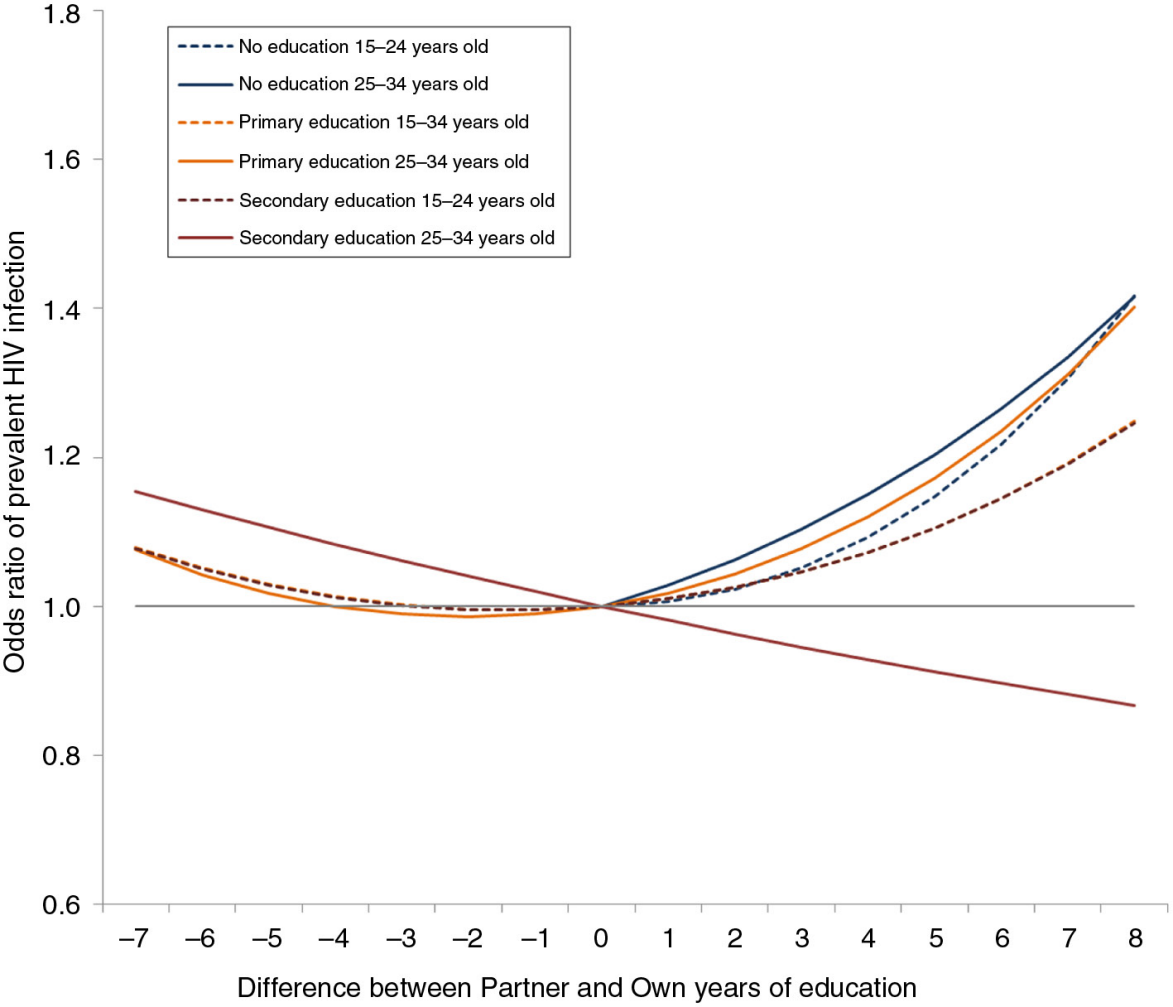
Association of relationship educational difference on woman's risk of prevalent HIV infection, stratified by woman's age. Odds ratios based on regression coefficients from a single model containing interactions of woman's educational attainment in levels and difference between partner and woman's education in years. Model also contains variables for urbanicity, country, survey round and woman's age (see Table 3, last two columns). Odds ratios calculated by combining linear and quadratic variable coefficients for each variable shown, and thus are relative to a woman in the same survey with the same education as their partner and in the same stratum of educational attainment.

In model 3, we included an interaction of educational difference and each woman's educational attainment level. Here, there was no evidence of heterogeneity across survey countries or timepoints; however, there was a clear gradient such that having a large educational difference was most strongly associated with HIV for women with no education and least strongly associated for those with secondary education or above. When we stratified this model by woman's age (Figure 3), educational difference continued to be associated with HIV prevalence in both age groups; however, the effect was homogenous across own education levels (*F*_(4, 5271)_=0.53, *p=*0.71) and weaker (*F*_(6, 5269)_=4.34, *p*<0.001) amongst women aged 15–24, while heterogeneous (*F*_(4, 6063)_=5.84, *p*<0.001) and stronger (*F*_(6, 6061)_=11.4, *p*<0.001) amongst those aged 25–34.

## Discussion

In this study, we examined the pattern of partnering by education in SSA, and evaluated whether differences in educational attainment within relationships were associated with HIV status. In line with prior evidence [30,34–36], we found that individuals partner assortatively with respect to education in SSA. The levels of assortativity were generally in the range 0.2–0.4, reflecting consistent like-with-like partnering at levels comparable to those seen educational assortativity in the United States [55] and in urban SSA [56]. Assortativity was lower (but still positive) when ceiling (e.g. urban Zimbabwe) or floor (e.g. rural Rwanda) effects reduced variability in education levels. Educational assortativity was associated with neither female educational attainment nor with HIV prevalence at the regional or national levels in these data. Such null findings suggest the absence of region-level social factors relating greater prevalence of socioeconomically unequal relationships with HIV rates, although there remains considerable scope to further investigate such associations for other social factors (e.g. age disparities, the interaction of age-disparity and educational difference) or lower levels of aggregation (e.g. town or district). It is also possible that non-marital relationships are more relevant for mixing between high- and low-HIV-prevalence populations and HIV acquisition, since partnership turnover is typically higher outside marriage.

While educational assortativity at the group level was not associated with prevalent HIV amongst women in SSA, there was a small but significant individual-level association between educational differences in relationships and women's likelihood of being HIV positive. The exact nature of this association varied by country. In 2004, in Ethiopia and Lesotho, higher levels of male education were linearly associated with higher likelihood of HIV infection; in 2008 in Kenya, this effect was reversed. However, in contrast to our initial hypothesis, HIV was most often associated with the absolute difference in educational attainment within marital relationships, rather than relative attainment levels. The finding that relationships which are educationally unequal – rather than containing a more-educated man – are positively associated with female HIV infections suggests that the association may reflect that relationship power differentials, rather than level of knowledge or income, are driving HIV risk.

We initially hypothesized that the association between educational differences and HIV would change over time, in line with evidence for women's education and HIV. While we once again found that the association between women's education and HIV is flattening over time in most countries (Figure 2a), we did not find a similar effect for educational differences (Figure 2b). This static result suggests that the association between educational difference and HIV is likely to reflect constant factors (e.g. income, power) rather than changing ones (e.g. HIV prevention knowledge).

In addition to geographic heterogeneity in the association between educational difference and HIV, we also saw heterogeneity based on a combination of woman's age and their educational attainment level. For women aged 15–24, larger absolute educational differences were consistently associated with prevalent HIV, most strongly if the woman had no education herself. For women aged 25–34, those with less than secondary education had a similar association, but more-educated women saw an almost linear decrease in risk with increasing partner education. This effect-modification of education difference by a combination of older age and higher own education suggests that those women with highest social status have a different risk profile for HIV infection. One explanation for this heterogeneity is that these high-status women are better able to negotiate safer behaviour within their relationships, offsetting the potentially risky aspects of a well-educated partner, such as higher mobility and income, which affords the man more opportunities to acquire HIV. An alternative reading could be that women who survive until older ages and attain more education have more sway in the marriage market and are able to select less-risky mates.

Finally, it is important to note that across the interquartile range of relationship educational differences seen in this dataset (man having three years more education than the woman vs. having the same level), the change in odds ratio is at most 12% in the 14 datasets. This figure is far lower than the changes in odds ratios across the interquartile range for women's own education (0–8 years). Thus, while education differences are associated with HIV risk, partner's education is likely to play a small additional role in predicting HIV risk for most women.

This study had a number of strengths. The analysis was based on nationally representative surveys conducted in a consistent manner across the seven countries involved, allowing for cross-country comparisons. The large sample sizes available provided power to detect effects and variation in effects.

Nevertheless, we also faced some limitations. First, our data were cross-sectional. This did not affect our ability to judge the benefits of current-partner's education in identifying those already infected with HIV, but meant we could not assess whether partner education is a risk factor for HIV acquisition. In addition, since we did not measure education prior to HIV infection, in some cases, HIV status may drive observed education differences. This reverse-causation process could occur if women who learn that they are HIV seropositive selectively seek out men better able to care for them (i.e. high status, more-educated partners), or conversely if women known to be seropositive face limited partnership opportunities, and thus partner with low status, low-education individuals. In addition, the lack of longitudinal data may mean that associations are driven by frailty effects, since those with higher levels of education are likely to be better able to access care, and thus to be more likely to be alive at the time of DHS interview.

Second, educational attainment (both of self and partner) is self-reported in the DHS. While reports of women's partners’ education are very highly correlated with the men's self-reported education, there may still be error in recall over time, leading to bias in our findings. Third, given the nature of the DHS datasets, we were unable to analyse the data from the perspective of men, to determine whether the findings we present for women are also true for men: since relationships are dynamic, there is no a priori reason to expect the effect of partner education status to be symmetric across genders. Such an analysis of men would therefore be a useful extension of our work. Fourth, a varying but substantial proportion of respondents declined to test for HIV in each survey, presenting the opportunity for selection bias. It is not clear, however, why non-participation should be correlated with the association between relationship educational difference and HIV status.

Fourth, while the DHS provides an opportunity to make comparisons across and within countries, it is important to consider carefully whether these comparisons are like-for-like. In this study, we consider educational attainment as a proxy measure for knowledge, cognition, income, and mobility; insofar as the same level of education provides differing benefits on any of these axes, years of education attained will necessarily be a noisy measure of the factors believed to drive HIV infection risk. Our finding of heterogeneous associations between educational differences and HIV across countries may reflect this heterogeneity in the meaning of educational attainment across space.

Finally, large sample size notwithstanding, we did not have sufficient power to estimate associations for every level of the interaction of women's educational attainment and educational difference: when we did so cell sizes were small and thus effect measures uncertain (Supplementary Figure 2).

## Conclusions

This analysis provides a first insight into the complex interactions of own and partner education in placing women at risk of infection with HIV in SSA. Future work to extend our findings could focus on other partner characteristics, on collecting longitudinal data to determine temporality of effects, and on collecting broader social and sexual network characteristics that would allow us to understand how a partner's education level does or does not place women at risk of HIV infection.

Our findings have implications for potential HIV prevention or mitigation interventions. If subsequent studies show that these associations reflect causal processes leading from partner education status to HIV, then interventions can be built which are tailored to the interrelationship between educational status of men and women. Such further analyses would need to determine whether any such causal link passes through differential take-up of protective knowledge within couples, differential application of this knowledge due to power imbalances or some other mechanism. Understanding of these mechanisms would allow interventions to be built that would reduce barriers within relationships, either by supporting knowledge transfer or empowering individuals to use their knowledge in practice.

Even without additional studies, this analysis suggests that targeting efforts to locate women infected with HIV, or at risk of HIV infection, should consider not only their own characteristics but also those of their sexual partners. This message fits within a broader message that successful interventions for HIV treatment and prevention will benefit from careful consideration of each social context [57,58], and of each person's place within it [59,60].

## Supplementary Material

The role of partners’ educational attainment in the association between HIV and education amongst women in seven sub-Saharan African countries

## References

[1] Tanser F, Bärnighausen T, Cooke GS, Newell M-L (2009). Localized spatial clustering of HIV infections in a widely disseminated rural South African epidemic. Int J Epidemiol.

[2] Hargreaves JR, Bonell CP, Morison LA, Kim JC, Phetla G, Porter JD (2007). Explaining continued high HIV prevalence in South Africa: socioeconomic factors, HIV incidence and sexual behaviour change among a rural cohort, 2001–2004. AIDS.

[3] Grabowski MK, Lessler J, Redd AD, Kagaayi J, Laeyendecker O, Ndyanabo A (2014). The role of viral introductions in sustaining community-based HIV epidemics in rural Uganda: evidence from spatial clustering, phylogenetics, and egocentric transmission models. PLoS Med.

[4] UNAIDS (2014). Issues brief: local epidemics.

[5] Potts M, Halperin DT, Kirby D, Swidler A, Marseille E, Klausner JD (2008). Reassessing HIV prevention. Science.

[6] Fox AM (2010). The social determinants of HIV serostatus in sub-Saharan Africa: an inverse relationship between poverty and HIV?. Public Health Rep.

[7] Gillespie S, Kadiyala S, Greener R (2007). Is poverty or wealth driving HIV transmission?. AIDS.

[8] Bärnighausen T, Hosegood V, Timaeus IM, Newell ML (2007). The socioeconomic determinants of HIV incidence: evidence from a longitudinal, population-based study in rural South Africa. AIDS.

[9] Hargreaves JR, Glynn JR (2002). Educational attainment and HIV-1 infection in developing countries: a systematic review. Trop Med Int Health.

[10] Wojcicki JM (2005). Socioeconomic status as a risk factor for HIV infection in women in East, Central and Southern Africa: a systematic review. J Biosoc Sci.

[11] Fortson JG (2008). The gradient in sub-Saharan Africa: socioeconomic status and HIV/AIDS. Demography.

[12] Cutler DM, Lleras-Muney A, Schoeni RF, House JS, Kaplan GA, Pollack H (2008). Education and health: evaluating theories and evidence. Making Americans healthier: social and economic policy as health policy.

[13] Jukes M, Simmons S, Bundy D (2008). Education and vulnerability: the role of schools in protecting young women and girls from HIV in southern Africa. AIDS.

[14] Hargreaves JR, Bonell CP, Boler T, Boccia D, Birdthistle I, Fletcher A (2008). Systematic review exploring time trends in the association between educational attainment and risk of HIV infection in sub-Saharan Africa. AIDS.

[15] Kayeyi N, Sandøy IF, Fylkesnes K (2009). Effects of neighbourhood-level educational attainment on HIV prevalence among young women in Zambia. BMC Public Health.

[16] Magadi M, Desta M (2011). A multilevel analysis of the determinants and cross-national variations of HIV seropositivity in sub-Saharan Africa: evidence from the DHS. Health Place.

[17] Magadi MA (2013). The disproportionate high risk of HIV infection among the urban poor in sub-Saharan Africa. AIDS Behav.

[18] de Walque D (2009). Does education affect HIV status? Evidence from five African Countries. World Bank Econ Rev.

[19] Gabrysch S, Edwards T, Glynn JR (2008). The role of context: neighbourhood characteristics strongly influence HIV risk in young women in Ndola, Zambia. Trop Med Int Health.

[20] Hargreaves JR, Davey C, White RG (2013). Does the'inverse equity hypothesis’ explain how both poverty and wealth can be associated with HIV prevalence in sub-Saharan Africa?. J Epidemiol Community Health.

[21] Gummerson E (2012). From Predictive to Protective? Is the relationship between HIV and education changing?.

[22] Hargreaves JR, Howe LD (2010). Changes in HIV prevalence among differently educated groups in Tanzania between 2003 and 2007. AIDS.

[23] Iorio D, Santaeulàlia-Llopis R (2011). Education, HIV status, and risky sexual behavior: how much does the stage of the HIV epidemic matter?.

[24] de Walque D, Nakiyingi-Miiro JS, Busingye J, Whitworth JA (2005). Changing association between schooling levels and HIV-1 infection over 11 years in a rural population cohort in south-west Uganda. Trop Med Int Health.

[25] Gummerson E (2013). Have the educated changed HIV risk behaviors more in Africa?. Afr J AIDS Res.

[26] Hargreaves JR, Slaymaker E, Fearon E, Howe LD (2012). Changes over time in sexual behaviour among young people with different levels of educational attainment in Tanzania. J Int AIDS Soc.

[27] Mishra V, Assche SB, Greener R, Vaessen M, Hong R, Ghys PD (2007). HIV infection does not disproportionately affect the poorer in sub-Saharan Africa. AIDS.

[28] Dunkle KL, Jewkes RK, Brown HC, Gray GE, McIntryre JA, Harlow SD (2004). Transactional sex among women in Soweto, South Africa: prevalence, risk factors and association with HIV infection. Soc Sci Med.

[29] Swidler A, Watkins SC (2007). Ties of dependence: AIDS and transactional sex in rural Malawi. Stud Fam Plann.

[30] Smits J, Ultee W, Lammers J (1998). Educational homogamy in 65 countries: an explanation of differences in openness using country-level explanatory variables. Am Sociol Rev.

[31] Mare RD (1991). Five decades of educational assortative mating. Am Sociol Rev.

[32] Smits J, Park H (2009). Five decades of educational assortative mating in 10 East Asian societies. Soc Forces.

[33] Huijts T, Monden CW, Kraaykamp G (2010). Education, educational heterogamy, and self-assessed health in Europe: a multilevel study of spousal effects in 29 European countries. Eur Sociol Rev.

[34] Esteve A, McCaa R (2008). Assortative mating patterns in the developing world. IUSSP Seminar on Changing Transitions to Marriage.

[35] Spell S, Anglewicz P, Kohler H-P (2012). Marriage as a mechanism: women's education and wealth in Malawi.

[36] Quisumbing AR, Hallman K (2003). Marriage in transition: evidence on age, education, and assets from six developing countries.

[37] Monden CW, van Lenthe F, De Graaf ND, Kraaykamp G (2003). Partner's and own education: does who you live with matter for self-assessed health, smoking and excessive alcohol consumption?. Soc Sci Med.

[38] Egeland GM, Tverdal A, Meyer HE, Selmer R (2002). A man's heart and a wife's education: a 12-year coronary heart disease mortality follow-up in Norwegian men. Int J Epidemiol.

[39] Skalická V, Kunst AE (2008). Effects of spouses’ socioeconomic characteristics on mortality among men and women in a Norwegian longitudinal study. Soc Sci Med.

[40] Spoerri A, Schmidlin K, Richter M, Egger M, Clough-Gorr KM, Puhan M (2014). Individual and spousal education, mortality and life expectancy in Switzerland: a national cohort study. J Epidemiol Community Health.

[41] Huijts T, Monden CW, Kraaykamp G (2010). Education, educational heterogamy, and self-assessed health in Europe: a multilevel study of spousal effects in 29 European countries. Eur Sociol Rev.

[42] Jaffe DH, Eisenbach Z, Neumark YD, Manor O (2006). Effects of husbands’ and wives’ education on each other's mortality. Soc Sci Med.

[43] Torssander J, Erikson R (2009). Marital partner and mortality: the effects of the social positions of both spouses. J Epidemiol Community Health.

[44] Laumann EO, Youm Y (1999). Racial/ethnic group differences in the prevalence of sexually transmitted diseases in the United States: a network explanation. Sex Transm Dis.

[45] Adimora AA, Schoenbach VJ (2005). Social context, sexual networks, and racial disparities in rates of sexually transmitted infections. J Infect Dis.

[46] Swartzendruber A, Zenilman JM, Niccolai LM, Kershaw TS, Brown JL, Diclemente RJ (2013). It takes 2: partner attributes associated with sexually transmitted infections among adolescents. Sex Transm Dis.

[47] Aral SO, Hughes JP, Stoner B, Whittington W, Handsfield HH, Anderson RM (1999). Sexual mixing patterns in the spread of gonococcal and chlamydial infections. Am J Public Health.

[48] Hancock EB, Manhart LE, Nelson SJ, Kerani R, Wroblewski JK, Totten PA (2010). Comprehensive assessment of sociodemographic and behavioral risk factors for *Mycoplasma genitalium* infection in women. Sex Transm Dis.

[49] Jewkes R, Dunkle K, Nduna M, Levin J, Jama N, Khuzwayo N (2006). Factors associated with HIV sero-status in young rural South African women: connections between intimate partner violence and HIV. Int J Epidemiol.

[50] Dellar RC, Dlamini S, Abdool Karim Q (2015). Adolescent girls and young women: key populations for HIV epidemic control. J Int AIDS Soc.

[51] Joint United Nations Programme on HIV/AIDS (UNAIDS) (2014). The Gap Report.

[52] Mishra V, Vaessen M, Boerma JT, Arnold F, Way A, Barrere B (2006). HIV testing in national population-based surveys: experience from the Demographic and Health Surveys. Bull World Health Organ.

[53] Newman MEJ (2003). Mixing patterns in networks. Phys Rev E.

[54] Bohl DD, McFarland W, Raymond HF (2011). Improved measures of racial mixing among men who have sex with men using Newman's assortativity coefficient. Sex Transm Infect.

[55] Doherty IA, Schoenbach VJ, Adimora AA (2009). Sexual mixing patterns and heterosexual HIV transmission among African Americans in the southeastern United States. J Acquir Immune Defic Syndr.

[56] Kenyon C, Colebunders R (2013). Birds of a feather: homophily and sexual network structure in sub-Saharan Africa. Int J STD AIDS.

[57] UNAIDS/WHO Working Group on Global HIV/AIDS and STI Surveillance (2013). Guidelines for second generation HIV surveillance: an update: know your epidemic.

[58] Wilson D, Halperin DT (2008). “Know your epidemic, know your response”: a useful approach, if we get it right. Lancet.

[59] Boerma JT, Gregson S, Nyamukapa C, Urassa M (2003). Understanding the uneven spread of HIV within Africa: comparative study of biologic, behavioral, and contextual factors in rural populations in Tanzania and Zimbabwe. Sex Transm Dis.

[60] Helleringer S, Kohler HP (2007). Sexual network structure and the spread of HIV in Africa: evidence from Likoma Island, Malawi. AIDS.

