# Supplementary material for: The role of partners’ educational attainment in the association between HIV and education amongst women in seven sub-Saharan African countries
Source: J Int AIDS Soc. 2016 Feb 19;19(1):20038. doi: 10.7448/IAS.19.1.20038 (PMC4762222; doi:10.7448/IAS.19.1.20038)
Supplement: The role of partners’ educational attainment in the association between HIV and education amongst women in seven sub-Saharan African countries [file JIAS-19-20038-s001.pdf]

Supplementary Material

For manuscript: The role of partners' educational attainment in the association between HIV and education amongst women in seven sub-Saharan African countries

Supplementary Table 1: Eligibility and testing rates for households selected for the included Demographic and Health Surveys

Survey wave	Proportion of sample households eligible for HIV test	Proportion of eligible Women tested	Proportion of eligible Men tested
Cameroon 2004	One in two	92%	90%
Cameroon 2011	One in two	94%	92%
Ethiopia 2005	One in two	83%	76%
Ethiopia 2011	All	89%	82%
Kenya 2003	One in two	76%	70%
Kenya 2008	One in two	86%	79%
Lesotho 2004	One in two	81%	68%
Lesotho 2009	One in two	94%	88%
Malawi 2004	One in three	70%	63%
Malawi 2010	One in three	91%	84%
Rwanda 2005	One in two	97%	95%
Rwanda 2010	One in two	99%	98%
Zimbabwe 2005	All	76%	63%
Zimbabwe 2010	All	80%	69%

When not all households are eligible for HIV testing, this is typically because only those households included in the male survey sample were asked to test for HIV.

Supplementary Table 2: Sample descriptive statistics by survey and urbanicity, Assortativity sample

	Cameroon		Ethiopia		Kenya		Lesotho	
	2004	2011	2005	2011	2003	2008	2004	2009
All observations (n)	4,982	6,674	6,397	7,866	3,593	3,651	2,764	3,065
Urbanicity								
Non-urban	49.8	51.2	87.9	79.5	75.3	73.6	79.5	69.4
Urban	50.2	48.8	12.1	20.5	24.7	26.4	20.5	30.6
Own Age								
15-19	16.0	12.8	13.1	11.8	10.2	6.2	11.0	10.1
20-24	30.9	29.5	26.8	27.6	29.6	29.5	31.7	30.0
25-29	28.9	32.6	34.3	36.2	32.3	34.3	30.4	31.5
30-34	24.3	25.2	25.8	24.4	28.0	30.1	26.8	28.4
Own Education (years) †	6 [0 - 8]	6 [1 - 9]	0 [0 - 1]	0 [0 - 3]	8 [5 - 9]	8 [6 - 10]	7 [6 - 9]	7 [6 - 9]
Partner Education (years) †	7 [3 - 10]	6 [3 - 11]	0 [0 - 4]	2 [0 - 6]	8 [7 - 11]	8 [7 - 12]	6 [3 - 8]	7 [4 - 9]
Partner - Own years of education †	1 [0 - 4]	1 [0 - 4]	0 [0 - 3]	0 [0 - 3]	1 [0 - 3]	0 [0 - 3]	-2 [-4 - 0]	-1 [-4 - 1]

	Malawi		Rwanda		Zimbabwe	
	2004	2010	2005	2010	2005	2010
All observations (n)	6,932	12,363	3,778	4,697	4,216	4,395
Urbanicity						
Non-urban	83.7	81.8	85.7	86.2	65.3	64.8
Urban	16.3	18.2	14.3	13.8	34.7	35.2
Own Age						
15-19	12.5	10.5	1.9	2.2	11.7	10.6
20-24	36.5	30.9	28.0	23.1	31.8	29.8
25-29	30.1	33.4	36.4	40.3	30.2	32.7
30-34	20.9	25.3	33.7	34.4	26.3	26.9
Own Education (years) †	4 [1 - 7]	5 [3 - 8]	4 [1 - 6]	4 [2 - 6]	8 [7 - 10]	10 [7 - 11]
Partner Education (years) †	7 [3 - 10]	7 [4 - 10]	5 [0 - 7]	4 [2 - 6]	10 [7 - 10]	11 [9 - 11]
Partner - Own years of education †	2 [0 - 4]	2 [0 - 4]	0 [-2 - 3]	0 [-2 - 2]	0 [0 - 2]	1 [0 - 3]

Figures are proportions unless otherwise noted; † denotes medians and interquartile ranges. Proportions and percentiles are survey weighted using the DHS sample weights for the female sample.

Supplementary Table 3: Sample descriptive statistics by country and urbanicity, HIV sample

	Cameroon				Ethiopia				Kenya			
	2004		2011		2005		2011		2003		2008	
	N	% HIV	N	% HIV	N	% HIV	N	% HIV	N	% HIV	N	% HIV
All observations (n)	2,439	8.3	2,980	6.3	2,770	1.8	7,448	2.5	1,487	10.8	1,644	9.2
Urbanicity												
Non-urban	49.0	5.5	51.0	4.3	88.5	0.8	79.7	1.1	74.6	9.4	73.9	8.7
Urban	51.0	11.0	49.0	8.3	11.5	9.3	20.3	7.9	25.4	15.1	26.1	10.5
Own Age												
15-19	16.1	3.8	12.5	3.0	14.0	0.8	11.9	0.2	10.5	7.6	5.1	10.3
20-24	30.5	8.0	29.5	4.5	26.4	2.2	27.8	1.1	28.8	11.0	30.0	7.4
25-29	28.9	10.5	32.7	8.4	33.9	2.1	36.0	3.2	31.6	12.4	37.1	10.9
30-34	24.4	9.0	25.4	7.1	25.7	1.5	24.3	4.0	29.1	10.2	27.8	8.7
Own Education †	6 [0 - 8]		6 [1 - 9]		0 [0 - 1]		0 [0 - 3]		8 [5 - 9]		8 [6 - 10]	
Partner Education †	7 [2 - 10]		6 [3 - 11]		0 [0 - 4]		2 [0 - 6]		8 [7 - 11]		8 [7 - 12]	
Partner - Own years of education †	1 [0 - 4]		1 [0 - 4]		0 [0 - 2]		0 [0 - 3]		1 [0 - 3]		0 [0 - 3]	

	Lesotho				Malawi			
	2004		2009		2004		2011	
	N	% HIV	N	% HIV	N	% HIV	N	% HIV
All observations (n)	1,217	32.5	1,531	30.8	2,439	8.3	2,980	6.3
Urbanicity								
Non-urban	78.2	28.5	71.5	28.2	49.0	5.5	51.0	4.3
Urban	21.8	47.0	28.5	37.4	51.0	11.0	49.0	8.3
Own Age								
15-19	10.3	13.8	9.7	8.0	16.1	3.8	12.5	3.0
20-24	30.4	27.2	31.2	24.9	30.5	8.0	29.5	4.5
25-29	29.2	40.0	29.5	33.6	28.9	10.5	32.7	8.4
30-34	30.2	37.1	29.6	41.6	24.4	9.0	25.4	7.1
Own Education †	7 [6 - 9]		7 [6 - 9]		6 [0 - 8]		6 [1 - 9]	
Partner Education †	6 [2 - 9]		7 [3 - 9]		7 [2 - 10]		6 [3 - 11]	
Partner - Own years of education †	-2 [-4 - 0]		-1 [-4 - 1]		1 [0 - 4]		1 [0 - 4]	

	Rwanda				Zimbabwe			
	2005		2010		2005		2010	
	N	% HIV	N	% HIV	N	% HIV	N	% HIV
All observations (n)	2,770	1.8	7,448	2.5	1,487	10.8	1,644	9.2
Urbanicity								
Non-urban	88.5	0.8	79.7	1.1	74.6	9.4	73.9	8.7
Urban	11.5	9.3	20.3	7.9	25.4	15.1	26.1	10.5
Own Age								
15-19	14.0	0.8	11.9	0.2	10.5	7.6	5.1	10.3
20-24	26.4	2.2	27.8	1.1	28.8	11.0	30.0	7.4
25-29	33.9	2.1	36.0	3.2	31.6	12.4	37.1	10.9
30-34	25.7	1.5	24.3	4.0	29.1	10.2	27.8	8.7
Own Education †	0 [0 - 1]		0 [0 - 3]		8 [5 - 9]		8 [6 - 10]	
Partner Education †	0 [0 - 4]		2 [0 - 6]		8 [7 - 11]		8 [7 - 12]	
Partner - Own years of education †	0 [0 - 2]		0 [0 - 3]		1 [0 - 3]		0 [0 - 3]	

Figures are proportions unless otherwise noted; † denotes medians and interquartile ranges. Proportions and percentiles are survey weighted using the DHS sample weights for the HIV sample.

Supplementary Figure 1: Plots of Newman assortativity coefficient for educational attainment between male and female partners, and mean educational attainment in strata defined by geographic region and urbanicity.

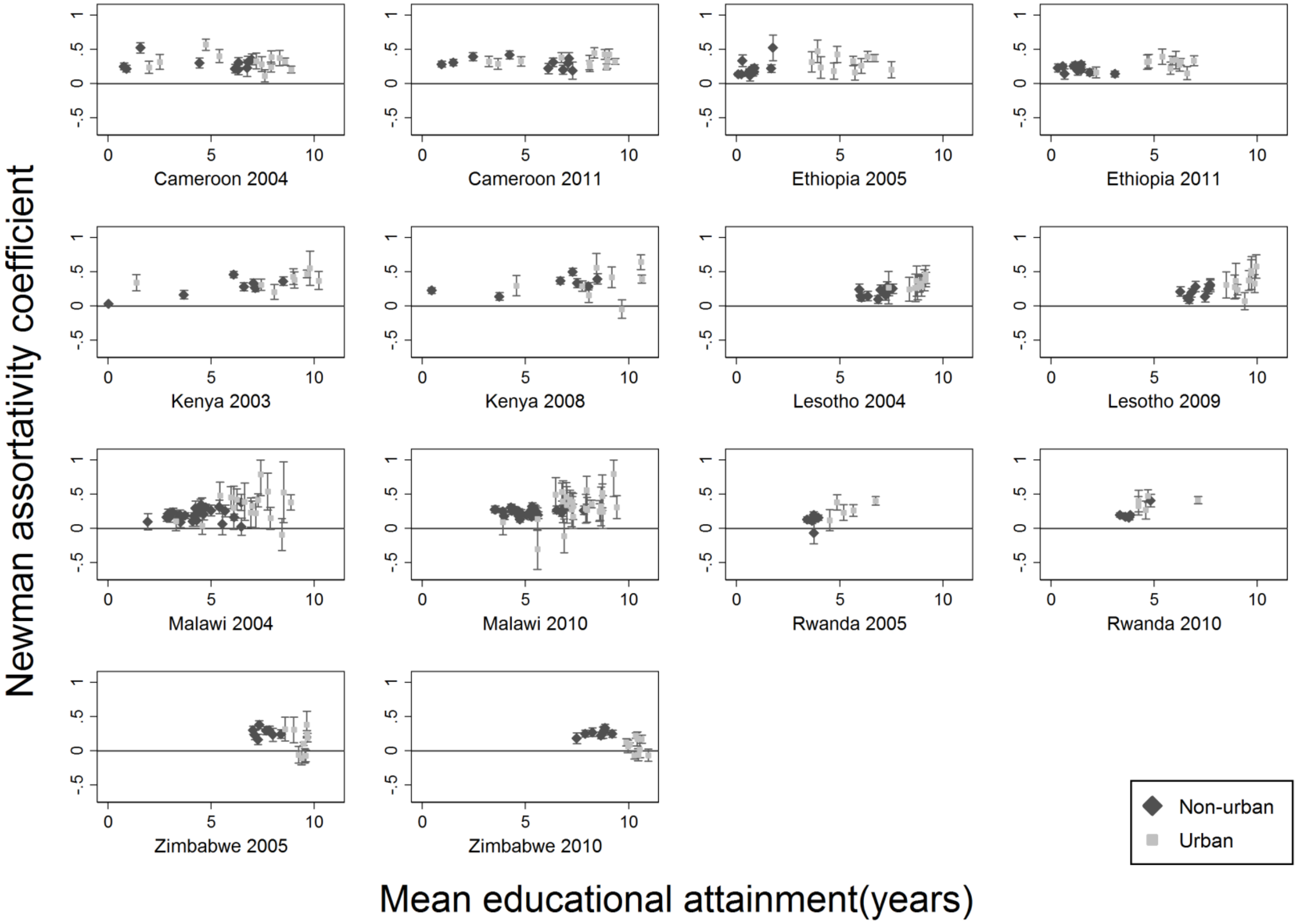

Partner-level educational assortativity was not correlated with mean female educational attainment (regionally, n=308: ρ=0.06, p=0.31; nationally, n=14: ρ=-0.01, p=0.97) using Pearson correlation coefficients. Sample size for this analysis was 75,373, weighted for the female sample.

Supplementary Figure 2: Comparisons of modelling approaches for regressions of prevalent HIV status in women on relationship educational attainment difference

In each subfigure below, the red line is the fitted values from the stratified versions of Model 3 in **Fel! Hittar inte referenskälla.** and **Fel! Hittar inte referenskälla.**. The blue points and confidence intervals are coefficient estimates from separate stratified regression models containing all the same covariates, but replacing the linear and quadratic terms with indicator variables for each year of education difference (and its interaction with woman's educational level). As can be seen, due to small cell sizes, estimates for specific years of educational difference are unstable with wide confidence intervals. We therefore used polynomial variables throughout our main analyses.

Fel! Hittar inte referenskälla. from the main text of the paper:

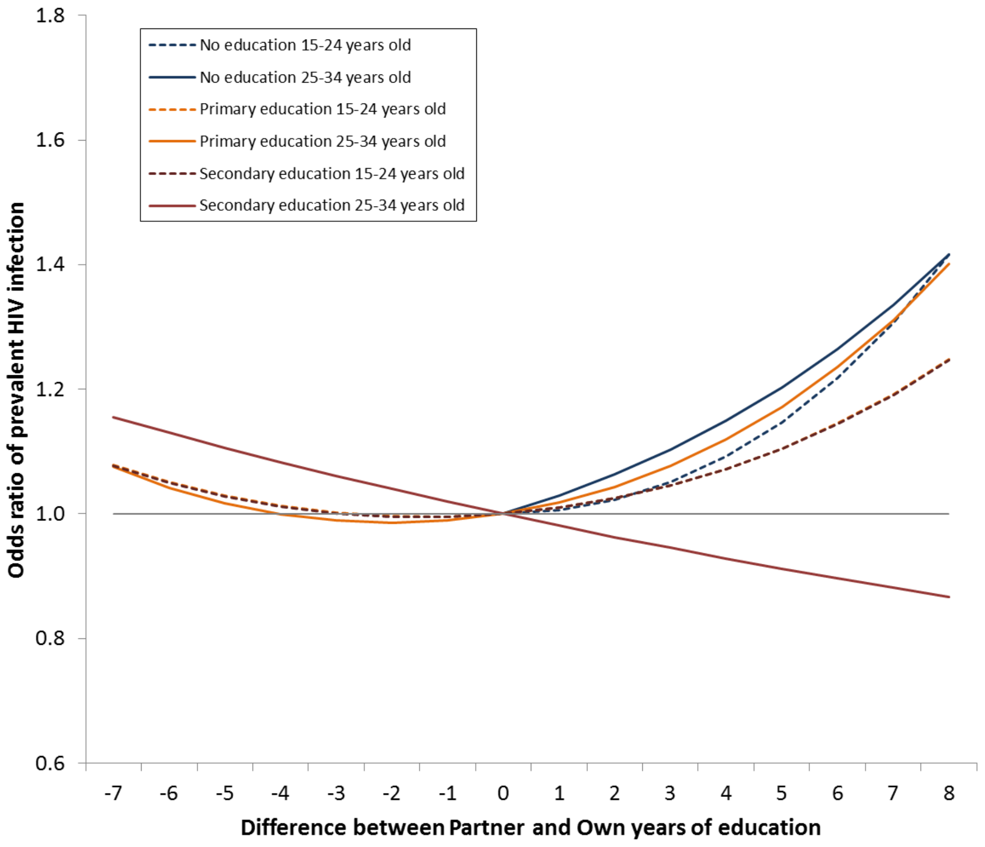

Comparison of single-year indicators for each of the six curves shown:

Figures for women with **no education**: A: aged 15-24; B: aged 25-34

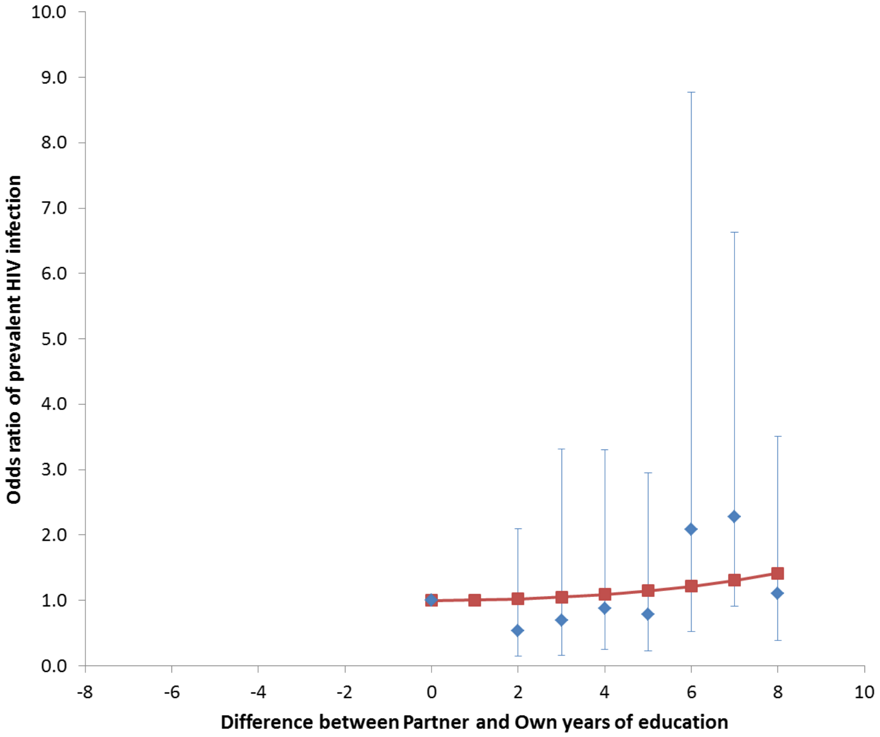

A.

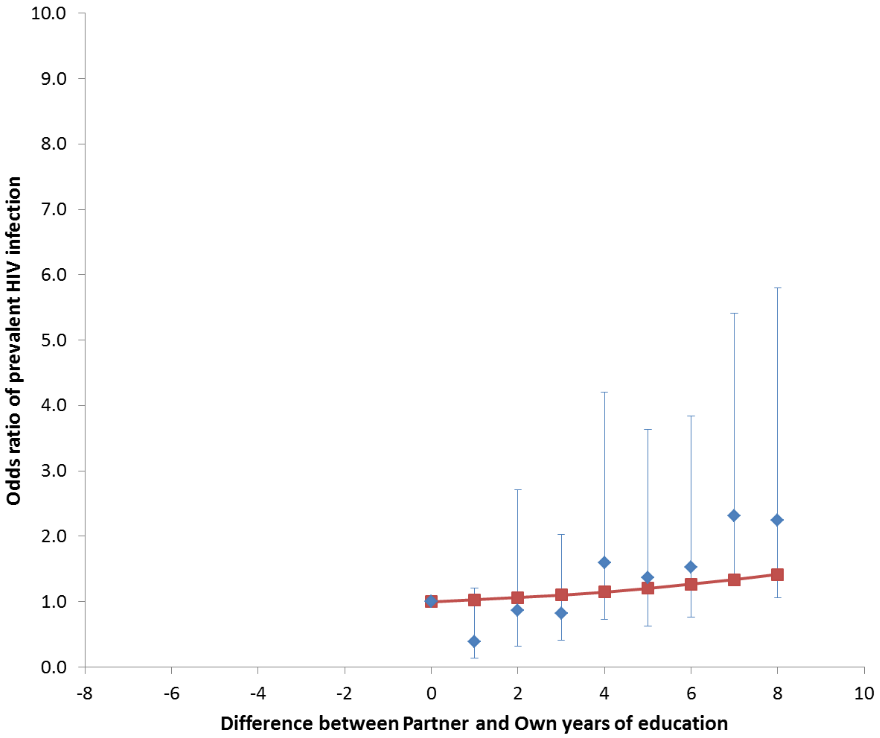

B.

Figures for women with **primary education**: C: aged 15-24; D: aged 25-34

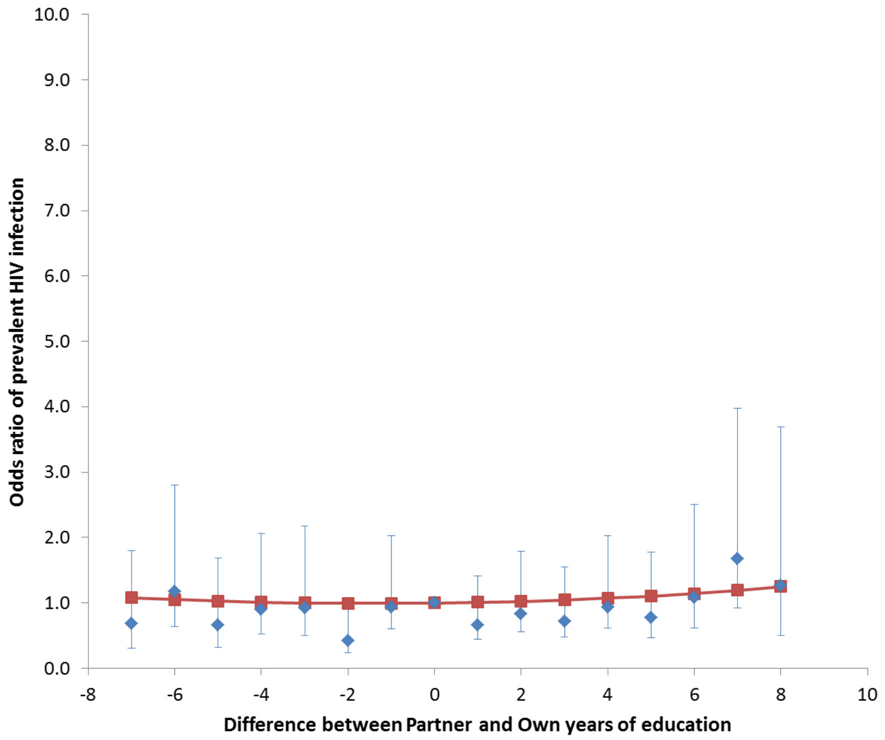

C.

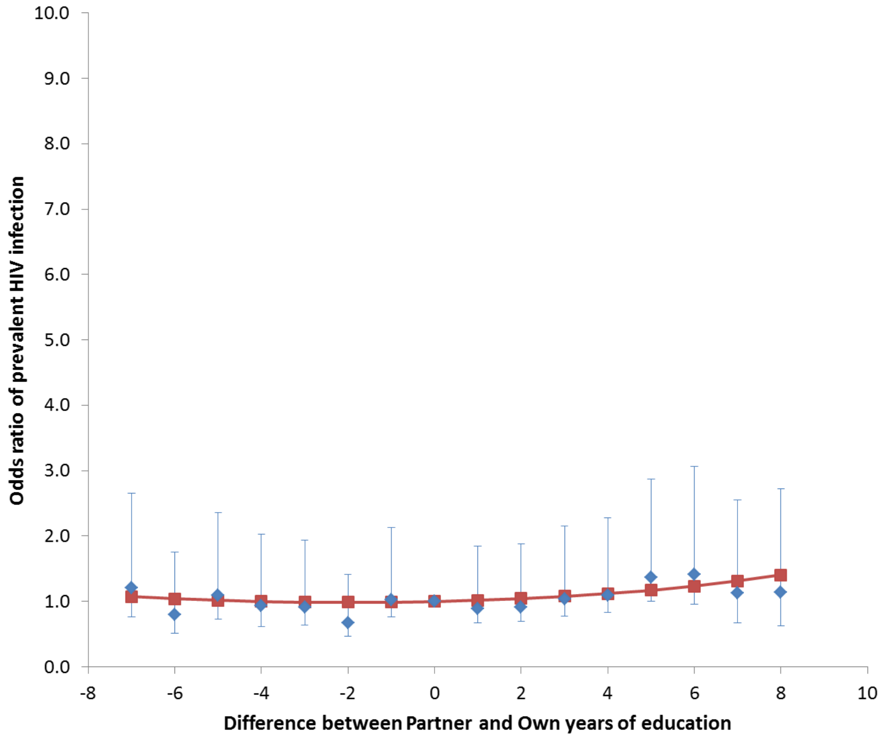

D.

Figures for women with **secondary or more education**: E: aged 15-24; F: aged 25-34

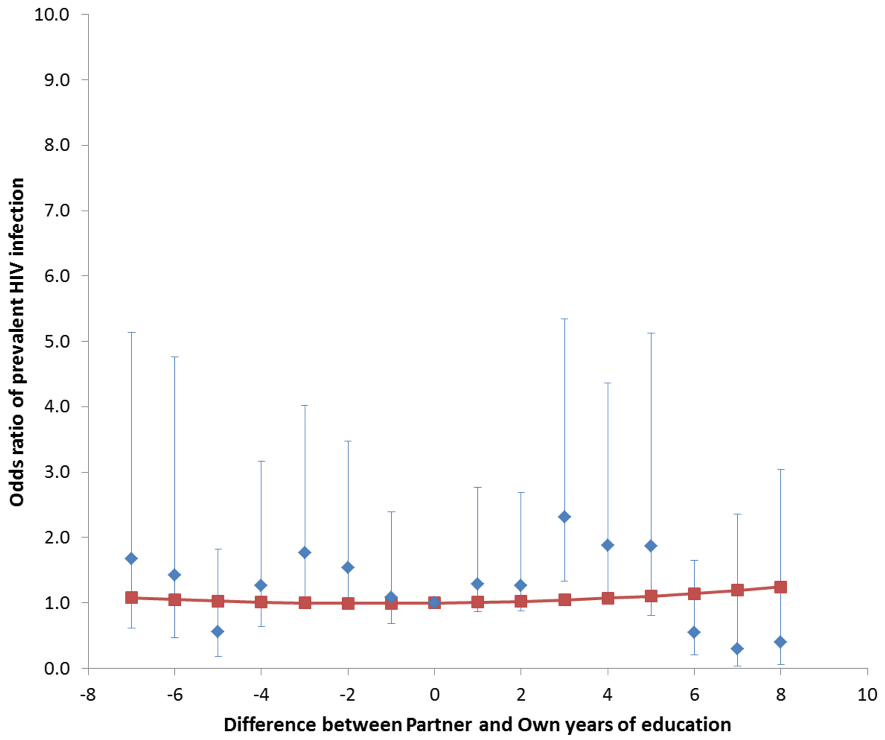

E.

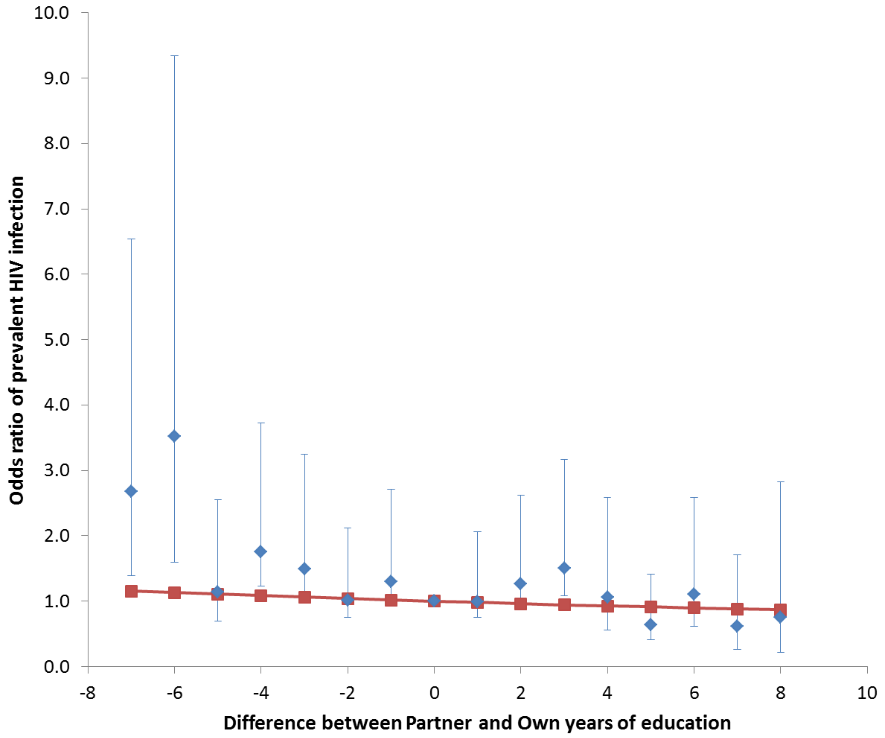

F.
